# Embryonal Carcinoma in Cryptorchid Abdominal Testis of an Infant

**DOI:** 10.1155/2015/383241

**Published:** 2015-06-07

**Authors:** Atia Zaka-ur-Rab, Zeeba Zaka-ur-Rab, Kafil Akhtar

**Affiliations:** ^1^Department of Surgery, Jawaharlal Nehru Medical College, Aligarh Muslim University, Aligarh 202002, India; ^2^Department of Pediatrics, Jawaharlal Nehru Medical College, Aligarh Muslim University, Aligarh 202002, India; ^3^Department of Pathology, Jawaharlal Nehru Medical College, Aligarh Muslim University, Aligarh 202002, India

## Abstract

Cryptorchidism is a known predisposing factor for the development of testicular tumors in adults. Age of patient at the time of treatment of undescended testes has some bearing on the risk of neoplasia. Testicular neoplasia at the time of primary surgery for cryptorchidism has been reported rarely in prepubertal period. We report a case where embryonal carcinoma was detected in a cryptorchid testis of an infant.

## 1. Introduction

Testicular tumors mostly present as a painless scrotal mass. These tumors are rarely encountered in the pediatric age group with the reported incidence being only 0.05–2 per 100,000 children [1]. Occurrence of germ cell tumor in infancy in a cryptorchid testis is still rarer with extensive search of literature revealing no such case. We report a case of embryonal carcinoma in a one-year-old infant with abdominal testis.

## 2. Case Presentation

A one-year-old male infant was admitted with the complaints of absence of right testis in the scrotum since birth and a lump in right lower abdomen for the last one and a half months. He was alert and playful with no other constitutional symptoms. The baby was a product of a nonconsanguineous marriage and had been delivered at term. His antenatal, birth, and development histories were unremarkable. On clinical examination, a nontender, firm, freely mobile, and oval lump measuring 6 × 4 cm was detected extending from right lumbar region to right iliac region. Right side of the scrotum was found to be smaller with no palpable testis. The left testis was normally descended with normal size, shape, and consistency. The external genitalia, except for the presence of cryptorchidism, were normal. No other congenital anomaly or facial dysmorphism was observed.

**Figure 1:**
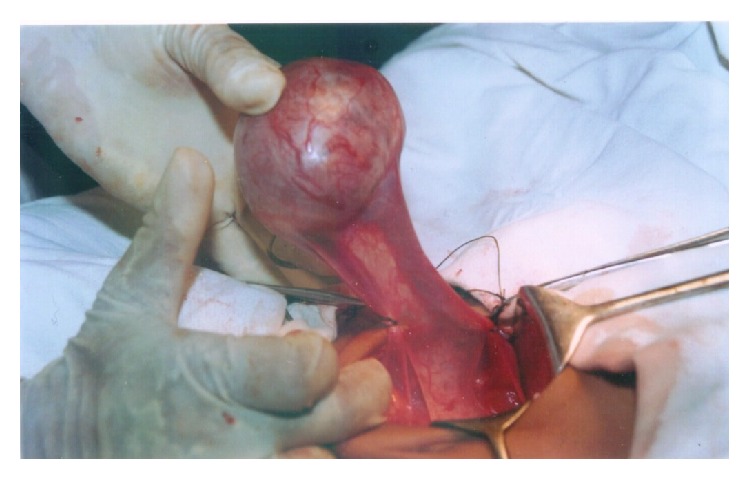
Per-operative photograph of cryptorchid testis.

Ultrasonographic evaluation of abdomen and scrotum revealed a well-defined encapsulated mass of a size of 64 × 44 mm (volume 91 cc) with solid and cystic components in right para-aortic region, just anterior to lower pole of right kidney. No free fluid in the peritoneal cavity or lymph node enlargement was seen. The scrotal sac was empty. Chest radiography was normal. Serum AFP and *β*-HCG levels were not elevated. Exploratory laparotomy revealed an intra-abdominal encapsulated right testicular tumor (Figure 1) without any enlargement of retroperitoneal lymph nodes. Radical orchiectomy was performed.

**Figure 2:**
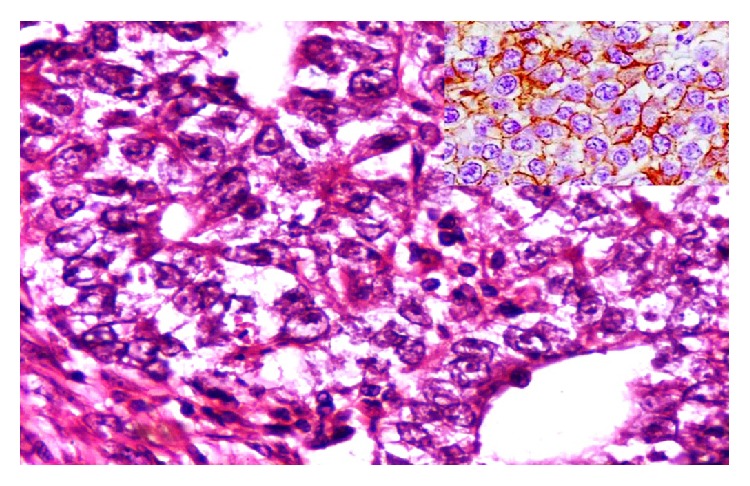
Micrograph showing nests of large pleomorphic typical epithelial cells with vesicular nucleus, prominent nucleoli, abundant cytoplasm, and poorly defined cell borders. H & E Stain × 40x. Inset: CD30 membranous positivity in the tumor cells IHC CD30 × 40x.

Histopathological examination of the resected specimen was suggestive of embryonal carcinoma with presence of nests of large pleomorphic typical epithelial cells with vesicular nucleus, prominent nucleoli, abundant cytoplasm, and poorly defined cell borders. Immunohistochemistry revealed CD30 membranous positivity in the tumor cells (Figure 2).

The patient was followed up regularly for 5 years after surgery. No evidence of recurrence was observed during this period.

## 3. Discussion

Cryptorchidism, which has long been recognized as a risk factor for testicular tumors in adults, is present in 2 to 4% of full-term male neonates. The prevalence decreases to about 1% by the age of 1 year [2]. Occurrence of testicular tumor in a cryptorchid testis was first described by Le Conte in 1851 [3]. As per a recent meta-analysis [4], boys with isolated cryptorchidism were three times more likely to develop testicular cancer.

The peak incidence of testicular cancer is observed in the third as well as fourth decades of life in both cryptorchids and noncryptorchids [5]. Testicular neoplasia at the time of primary surgery for cryptorchidism has been reported rarely in prepubertal period [6], with the risk being 5.2% in patients with intra-abdominal testis, abnormal external genitalia other than cryptorchidism, or diagnosed abnormal karyotype. At all ages, carcinoma in situ in undescended testis is considered a premalignant condition. Since testicular cancer develops very rarely in absence of male sex hormones, it is postulated that these hormones may play an important role in the transformation of carcinoma in situ of the testis into invasive cancer [7].

An abdominal cryptorchid testis is more likely to develop germ cell tumor than an inguinal testis [8]. Factors like altered environment, hormonal imbalance and testicular dysgenesis, or atrophy have all been implicated as possible oncogenic factors. Patients undergoing orchiopexy after age of 12 years or no orchiopexy were 2 to 6 times as likely to have testicular cancer as those who underwent prepubertal orchiopexy [9]. Even if orchiopexy was performed at an early age, the risk of testicular cancer still remained higher in these subjects as compared to those with normal testicular descent [10]. A large cohort study reported that the increased risk of testicular cancer in cryptorchids did not vary with the subject's age at surgery [11].

The most common histological types of testicular tumors reported in childhood are yolk sac tumors [12]. However, microscopically, over half of germ cell tumors consist of more than one cell type, requiring appropriate sampling for the correct diagnosis and correlation with the serum tumor markers [13]. Unlike in adults, human chorionic gonadotropin (HCG) does not serve as a helpful tumor marker for testicular tumors in the prepubertal population. However, raised levels of *α*-fetoprotein (AFP), observed in 90% of patients with yolk sac tumors, can help distinguish yolk sac tumors from other tumors [14]. On the other hand, AFP is only rarely positive in scattered embryonal carcinoma cells and helps in distinguishing yolk sac areas. Embryonal carcinomas are typically positive for placental alkaline phosphatase, c-kit (CD117), keratins (8, 18, and 19), and CD30. These tumors may also test positive for other markers, namely, OCT3/4, NANOG, SOX2 (sex-determining region Y [SRY)-box 2, and OCT3/4. Seminomas are negative for CD30 and SOX2 [15–18].

The majority (approximately 80%) of prepubertal patients with testicular tumors have clinical stage I disease (limited to the testis and completely excised) [14]. Recent studies suggest that stage I tumors are best managed with orchiectomy and surveillance that should include frequent thoracic and abdominal imaging and measurement of AFP levels [14, 19]. With observation alone, the recurrence rate for these patients is reported to be approximately 20% [13]. Nearly, all patients who develop metastatic or recurrent disease do so within 2 years [20]. Patients with recurrent disease or metastases at presentation can expect excellent results with platinum-based multiagent chemotherapy [14, 19]. Retroperitoneal lymph node dissection needs to be restricted to patients with persistent retroperitoneal masses following chemotherapy [14].

We wish to emphasize through this case report that even though the occurrence of germ cell tumor in infancy in cryptorchids is a rare phenomenon, the possibility of this condition should always be considered in all cases that present with a lump in the abdomen in association with undescended testes.
